# Pre-operative methicillin resistant *Staphylococcus aureus* results do not predict surgical site infections in children undergoing varus derotational osteotomy

**DOI:** 10.1097/MD.0000000000020517

**Published:** 2020-06-26

**Authors:** Alexander Nazareth, Sukhraj S. Bains, Lindsay M. Andras, Rachel Y. Goldstein, Robert M. Kay

**Affiliations:** a Keck School of Medicine, University of Southern California; b Children's Orthopaedic Center, Children's Hospital Los Angeles, Los Angeles, CA, USA.

**Keywords:** femoral varus derotational osteotomy, methicillin resistant *Staphylococcus aureus*, pre-operative screening, surgical site infections

## Abstract

Literature regarding the value of pre-operative nasal methicillin resistant *S*taphylococcus *aureus* (MRSA) swabs to predict surgical site infections (SSIs) in children undergoing lower extremity surgery is limited. The purpose of our study was to determine if pre-operative nasal MRSA swab results were predictive of SSI development in children undergoing a femoral varus derotational osteotomy (VDRO).

Patients who underwent VDRO between 2004–2016 were reviewed to determine pre-operative MRSA colonization rates and SSI devolvement rates. Patients with less than 1 year of follow-up, previous history of infections, or absent pre-operative MRSA swab were excluded. SSI rates of patients with negative MRSA and positive MRSA swab result were compared using the Fisher exact test. Aside from contact isolation precautions, no other changes in treatment were made during inpatient hospital course based on positive pre-operative nasal MRSA swab results.

247 patients met the inclusion criteria (mean age: 9.3 ± 3.6 years, 62% male). There were 242 (98%) patients with a negative MRSA swab and 5 (2%) patients with a positive MRSA swab. Out of the 242 patients with a negative MRSA swab, 4 developed an SSI. Of the patients with positive MRSA swab results, 0% (0/5) developed an SSI compared to 1.7% (4/242) of negative MRSA swab results who developed an SSI. Results indicated no significant difference in SSI development rates between the groups (*P* = 1.00).

In this series of children undergoing VDRO surgery, the results of a pre-operative MRSA nasal swab had no relationship to SSI incidence and no impact on clinical patient care. Pre-operative MRSA nasal swabs appear to be of limited benefit for routine pre-operative screening in this patient population.

Level III, retrospective comparative

## 1 Introduction

Surgical site infections (SSIs) are the most common nosocomial infection that occurs in the early post-operative period in surgical patients. Specifically, in orthopaedic patients, SSIs have been found to extend hospital stays, increase hospitalization and re-operation rates, and cost hospitals on average $860,000 per year.^[9,16]^ SSIs are most commonly caused by *Staphylococcus aureus* with an increasing prevalence of methicillin resistant *S aureus* (MRSA).^[3]^ Recent Center for Disease Control guidelines advocate the need for surveillance of epidemiologically important organisms including MRSA in high risk populations, facilities and procedures.^[5]^ California Health and Safety Code mandates MRSA screening within 24 hours of hospital admission, which is typically administered immediately prior to surgery at our institution.^[1]^

Intranasal swabs are commonly used to test for MRSA prior to inpatient surgical procedures, although the rate of MRSA nasal colonization has been reported to be less than 1% in the U.S. population.^[11]^ The World Health Organization recommends decolonization with mupirocin prior to orthopaedic procedures in adult patients colonized with MRSA; no recommendations were made regarding the pediatric population.^[2]^ Previous studies have reported conflicting results regarding the relationship between MRSA nasal colonization and subsequent SSIs.^[6,10,13,15]^ Current literature regarding the value of pre-operative nasal MRSA swabs to predict SSIs in children undergoing lower extremity surgery is limited. The purpose of our study was to determine if pre-operative nasal MRSA swab results were predictive of SSI in children undergoing a femoral varus derotational osteotomy.

## 2 Methods

Institutional Review Board approval was obtained for all study procedures before initiation of the study. Clinical data were retrospectively reviewed for children (age < 18) who underwent varus derotational osteotomy (VDRO) performed at the authors’ institution between 2004 and 2016. Patients were eligible if they underwent VDRO surgery and had a minimum of 1-year follow-up available within the study period. Patients who did not have a pre-operative nasal MRSA swab and those with insufficient clinical data were excluded. 247 patients met inclusion criteria (mean age at surgery: 9.3 ± 3.6 years, 62% male). Diagnoses included Cerebral Palsy (N = 205), hip dysplasia (N = 28) and other neuromuscular conditions (N = 14).

Pre-operative nasal MRSA swabs are administered at our institution as part of routine hospital procedure and tested at the on-site hospital lab. Nasal MRSA swabs were obtained on the day of surgery in the operating room with the child under general anesthesia using standard sterile swabs collected from the anterior nares. These specimens were subsequently cultured onto a chromogenic medium and monitored for growth via standardized microbiology lab practice guidelines. Due to our institution's policy and California mandated guidelines, MRSA swabs cannot be taken during the pre-operative visit and must therefore be taken on the day of surgery. Patient charts were retrospectively reviewed for demographic data including age, sex, diagnosis, MRSA swab results, presence of SSI and associated etiology (infecting organism). For those with a nasal swab positive for MRSA, any resultant changes in care based on the swab results were recorded. Patients routinely received ancef prior to incision which was continued postoperatively for 24 hours. In patients with an allergy preventing ancef administration clindamycin was typically given.

SSI rates were compared between patients with a negative versus a positive swab result using Fisher's exact test. All statistical analyses were performed in STATA (version 14.0, StataCorp LP, College Station, TX).

## 3 Results

247 patients met inclusion criteria (mean age at surgery: 9.3 ± 3.6 years, 62% male). Diagnoses included Cerebral Palsy (N = 205), hip dysplasia (N = 28) and other neuromuscular conditions (N = 14). There were 242 (98.0%) patients with a negative MRSA swab and 5 (2.0%) patients with a positive MRSA swab. 4 patients (1.6%) developed a SSI, all of which were MRSA swab negative patients. Of the 4 patients with SSIs, 2 were treated successfully with oral antibiotics, and the other 2 required surgical irrigation and debridement. For the 2 patients who were operatively debrided, the organisms identified were MSSA in 1 case and both MSSA and Pseudomonas aeruginosa in the other case. For the 2 patients who were treated with oral antibiotics, superficial swabs were taken and the organisms identified were MSSA in 1 case and only normal skin flora in the other case. There was no significant difference in SSI development rates between patients whose MRSA swab were positive and those who were negative (*P* = 1.00). There were no SSI patients with MRSA identified as the causative organism. No changes in treatment were made during inpatient hospital course based on positive nasal MRSA swab results, other than isolation precautions being instituted for those with positive MRSA swab results identified before hospital discharge.

## 4 Discussion

The MRSA nasal swab is routinely used as a pre-operative screening method based on the belief that results will influence treatment management and decrease SSI rates. This study was conducted to determine if the MRSA swab is a predictor of post-operative SSIs after VDRO surgery. Pre-operative screening demonstrated a 2% MRSA nasal colonization rate and the results of a pre-operative MRSA nasal swab had no relationship to SSI incidence in patients undergoing VDRO surgery. While many hospitals around the country have included an MRSA swab as part of their preoperative visit and instituted protocols for decolonization based on the results, due to our state and hospital policies these are obtained on the day of surgery. While this has hindered any opportunity to decolonize patients who are positive on the MRSA swab prior to surgery as the results are not obtained until after the procedure, it provides a unique opportunity to evaluate whether MRSA swabs truly have any impact on SSI rates in these cases.

In this series, preoperative screening demonstrated a 2% MRSA nasal colonization rate consistent with reports in the literature.^[13,14]^ Previous studies have demonstrated higher MRSA nasal colonization rates in previously hospitalized children and those with chronic conditions.^[7,12]^ Although many patients included in our study had complex medical histories requiring multiple previous hospitalizations we did not find a higher nasal colonization rate than reported in the literature for the general population. Due to the limited number of positive MRSA nasal swabs, we were unable to determine if baseline characteristics such as age at surgery, diagnosis, or sex affected colonization rates.

The results of a pre-operative MRSA nasal swab had no relationship to SSI incidence in patients undergoing VDRO surgery. Furthermore, pre-operative nasal MRSA screening did not change treatment in the 5 patients with a positive swab, except that these patients were placed on contact precautions as per hospital policy. Recent studies have found decreased MRSA infection rates to be correlated to decreasing patient to patient transmission.^[4,8]^ Therefore, although the patient with the positive MRSA swab may not have had any management changes from MRSA screening, it may be beneficial to identify these patients and place them on contact precautions to prevent transmission to other patients during their postoperative care. At our institution, MRSA swabs cost an average of $60 and the cost of screening our patient cohort was approximately $15,000.

Although this is a series of 247 VDRO patients, a relatively low rate of both MRSA colonization and SSI limited the ability to perform an analysis of these subgroups or identify characteristics that were significant risk factors. Previous studies on MRSA infections in other patient populations have reported similar limitations due to the low rates of colonization and SSI. Future larger scale multi-center and epidemiology based studies are needed to better elucidate the relationship between nasal colonization with MRSA and SSI rates.

In addition, there were a significant number of patients without nasal swab testing who were excluded that may have biased our results. Furthermore, it is possible that patients included in our study may have presented to an outside facility with an SSI which would not be captured in the retrospective chart review. However, we attempted to control for this by excluding patients with less than 1 year of follow-up after surgery. Also, it is possible that some patients included were intermittent colonizers and therefore may have tested negative with a nasal swab administered at a single time point. Furthermore, it is possible that there could have been false negative swabs which would have artificially lowered the MRSA colonization rates.

In conclusion, in this series of 247 children undergoing VDRO surgery the results of a pre-operative MRSA nasal swab had no relationship to SSI incidence and clinical care was not changed in any patient. Therefore, pre-operative MRSA nasal swabs appear to be of limited benefit for routine pre-operative screening in this patient population for SSI prevention. The utility of this pre-operative screening tool should be weighed against its cost (approximately $60 per patient and $15,000 in this series of patients).

## Author contributions

Alexander Nazareth: Data collection, data analysis, manuscript preparation

Raj Bains: Data collection

Lindsay M. Andras: Study Idea, study design, manuscript review

Rachel Y. Goldstein: Study Idea, study design, manuscript review

Robert M. Kay: Study idea, study design, manuscript review
